# Metabolic preference assay for rapid diagnosis of bloodstream infections

**DOI:** 10.1038/s41467-022-30048-6

**Published:** 2022-04-28

**Authors:** Thomas Rydzak, Ryan A. Groves, Ruichuan Zhang, Raied Aburashed, Rajnigandha Pushpker, Maryam Mapar, Ian A. Lewis

**Affiliations:** 1 Department of Biological Science, University of Calgary, Calgary, AB T2N 1N4 Canada; 2 Biomedical Engineering, University of Calgary, Calgary, AB T2N 1N4 Canada

**Keywords:** Applied microbiology, Clinical microbiology, Infectious-disease diagnostics

## Abstract

Bloodstream infections (BSIs) cause >500,000 infections and >80,000 deaths per year in North America. The length of time between the onset of symptoms and administration of appropriate antimicrobials is directly linked to mortality rates. It currently takes 2–5 days to identify BSI pathogens and measure their susceptibility to antimicrobials – a timeline that directly contributes to preventable deaths. To address this, we demonstrate a rapid metabolic preference assay (MPA) that uses the pattern of metabolic fluxes observed in ex-vivo microbial cultures to identify common pathogens and determine their antimicrobial susceptibility profiles. In a head-to-head race with a leading platform (VITEK 2, BioMérieux) used in diagnostic laboratories, MPA decreases testing timelines from 40 hours to under 20. If put into practice, this assay could reduce septic shock mortality and reduce the use of broad spectrum antibiotics.

## Abstract (web summary)

It is currently slow to identify bloodstream infection pathogens. Here the authors report a rapid metabolic preference assay that uses the pattern of metabolic fluxes observed in ex-vivo microbial cultures to identify common pathogens and determine their antimicrobial susceptibility profiles.

## Introduction

**Fig. 1:**
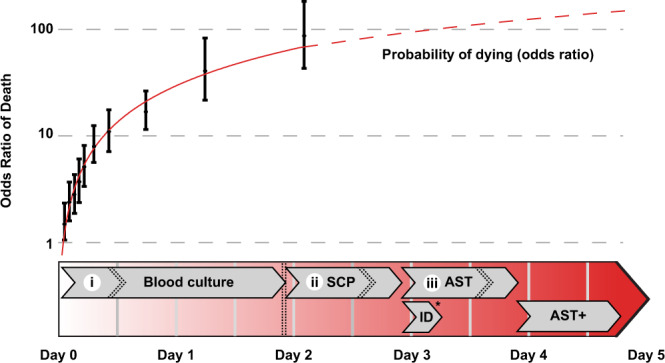
Bloodstream infection (BSI) testing timelines versus mortality rates. Mortality risk (expressed as adjusted odds ratio of death) adapted with permission from Kumar et al., 2006^2^ shown between the onset of symptoms and administration of antimicrobials (top) relative to current clinical testing times (bottom). Error bars represent the 95% confidence interval of the odds ratio. ID, microbial identification by MALDI-TOF-MS (matrix-assisted laser desorption ionization time of flight mass spectrometry); SCP single colony purification on agar plates, AST antimicrobial susceptibility testing, AST+ additional antimicrobial susceptibility testing for isolates with an unusual resistance profile. Roman numerals represent the rate-limiting microbial culturing steps. Dashed lines show the median culture timelines observed in high-volume diagnostic laboratories whereas the solid lines show the range of analysis times for each step. *While ID can be performed either after SCP or directly from blood culture bottles, the need for SCP for current AST methods means that overall timelines are unaffected. Error bars indicate a 95% confidence interval.

The diagnostic tools used to identify pathogens and measure antimicrobial susceptibility play a critical role in controlling infectious diseases. In the case of bloodstream infections (BSIs), rapid diagnostic timelines are critical because a patient’s odds of surviving an infection are inversely proportional to the length of time that elapses between the onset of symptoms and the administration of appropriate antimicrobials^1,2^. A single day of receiving an ineffective antimicrobial increases average BSI mortality by up to 5%^3,4^. When BSIs progress into septic shock, these treatment delays can increase mortality by over 7% per hour (Fig. 1)^2^. Unfortunately, most diagnostic laboratories require 2–5 days to complete microbial identification (ID) and antimicrobial susceptibility testing (AST; Fig. 1). Currently, 20–30% of patients are prescribed the wrong antimicrobial treatment^5,6^, a percentage that is expected to rise with the increasing prevalence of antimicrobial-resistant organisms. Given that 1.3 million people suffer from BSIs in North America and Europe each year, and roughly 182,000 of these people die^7^, a more efficient method for completing ID and AST could save tens of thousands of lives each year, decrease in-patient hospital stays, and decrease average treatment costs^8,9^. In addition, long diagnostic times contribute to the selection of antimicrobial-resistant organisms^10^ because the time-sensitive nature of treating BSIs forces clinicians to make therapeutic decisions with little or no laboratory data. This situation encourages the widespread use of broad-spectrum antimicrobials and selection for resistant organisms^11^. In summary, any technology that shortens the diagnostic timeline will have a significant impact on global health^1,12,13^.

The current clinical microbiology testing timeline is dominated by three microbial culturing steps that are necessary for identifying and characterizing bloodstream pathogens (Fig. 1). These microbial amplification steps are the rate-limiting factors in the analysis pipeline and are the primary technical roadblocks to faster diagnostics. In the first culturing step (i) patient blood samples are incubated for 7–43 h^14^ to allow microbes present in the samples to grow to detectable densities (from 0.01–100 CFU/mL to >1 × 10^9^ CFU/mL^15,16^). Although the median time to positivity is 12.7 h, this step can require as much as 125 h for some slow-growing organisms^17,18^. Once blood cultures have flagged positive, (ii) aliquots of cultures are streaked onto agar plates and incubated for another 12–24 h^19^ to obtain single colonies. These subcultured isolates are then identified via matrix-assisted laser desorption/ionization time-of-flight (MALDI-TOF) mass spectrometry (MS)^20^. Antimicrobial susceptibility testing is then performed by (iii) incubating a fixed number of microbes (5 × 10^5^ CFU/mL) for 12–24 h in a medium containing antimicrobials using an automated testing system (e.g., VITEK 2, BioMérieux; MicroScan, Beckman Coulter; Sensititre, Thermo Fisher Scientific; Phoenix, BD)^21^. Additional antimicrobial testing procedures may also be necessary for isolates with an unusual resistance profile^22^. In summary, current clinical diagnostic timelines are limited by microbial growth rates.

Both cost constraints and health concerns have created considerable pressure to develop a faster clinical diagnostic pipeline^23^. Some time savings have been achieved by streamlining the existing workflow. Direct MALDI-TOF-MS analysis of blood cultures, for example, allows microbes to be identified faster by circumventing one microbial culture step (Fig. 1ii)^24^. Unfortunately, direct MALDI-TOF-MS cannot replace the existing AST workflow. An alternative emerging strategy has been to use DNA-based technologies to both identify organisms and detect common resistance genes in a single multiplexed assay (e.g., Biofire® FilmArray® and Verigene®^25^). Although promising, these assays have limitations: they require culture-based isolation of the pathogen, are susceptible to false-negative results due to primer specificity or PCR inhibition, and can give false-positive results due to cell-free DNA^26,27^. Moreover, both proteomic and DNA-based assays detect the genetic potential for drug resistance, not the empirically determined antimicrobial susceptibility phenotype^28^. Any genetic modulators of resistance, or novel resistance mechanisms, cannot be detected via these assays. Consequently, direct phenotypic assessment of susceptibility via microbial culturing is required by the current clinical laboratory guidelines^22^.

Although faster molecular-based diagnostic tools are emerging, the need to both identify organisms and empirically determine their antimicrobial resistance profiles has prevented many of these tools from being integrated into working diagnostic laboratories. Metabolomics offers a unique opportunity to accelerate diagnostics while conforming to the established workflow used in clinical practices. Secreted metabolites can be thousands of times more abundant than individual proteins^29^, are sensitive reporters of microbial physiology^30^, and are compatible with established high-throughput clinical mass spectrometry platforms^31^. As a result, sensitive metabolite-based assays have the potential to minimize the rate-limiting steps in the existing clinical workflow. Although the applicability of metabolomics to microbial diagnostics has been recognized for over a decade^32–37^, previous applications have largely sought to identify native biomarkers present in the blood of people with infections. This approach is challenging due to the intrinsic variability of human metabolism. Moreover, it does not provide a mechanism for assessing antimicrobial susceptibility.

Herein, we introduce a diagnostic strategy, the metabolic preference assay (MPA), that uses the patterns of consumed versus excreted metabolites of ex vivo microbial cultures to both identify pathogens and measure their antimicrobial susceptibility. We identify biomarkers capable of differentiating seven of the most prevalent organisms responsible for BSIs [*Candida albicans* (CA), *Klebsiella pneumoniae* (KP), *Escherichia coli* (EC), *Pseudomonas aeruginosa* (PA), *Staphylococcus aureus* (SA), *Enterococcus faecalis* (EF), and *Streptococcus pneumoniae* (SP)]^38^, show that changes in metabolite levels using the metabolic inhibition assay (MIA) can empirically measure the activity of antimicrobials, and demonstrate that the MPA/MIA can produce results in a fraction of the time as current testing methods.

## Results

### Seven metabolites can differentiate between prevalent species responsible for of BSIs

Although the core architecture of central carbon metabolism is shared among almost all organisms, the activities of these pathways differ according to both genetic and environmental factors. Thus, by tightly controlling environmental variables, metabolic pathway activities should serve as indicators of microbial species. This fundamental hypothesis is the root of the MPA-based diagnostic approach and is testable by quantifying metabolic boundary fluxes (the rates at which nutrients and waste products are consumed or produced) of clinically relevant microbes under well-controlled conditions. To test this, the metabolic boundary fluxes of the seven most common bloodstream pathogens were measured. For initial biomarker discovery, three independent experiments were performed (*n* = 3), where three clinical isolates (2 for PA) of each target species (*n* = 7) were incubated independently in triplicate (*n* = 3) and analyzed. Microbial cultures were seeded at a 0.5 McFarland (OD_600_ ~0.15 or 7.5 × 10^7^ CFU/mL) into Mueller Hinton broth containing 10% human blood (MHB). Metabolite levels present in the cultures were analyzed at 0 h and 4 h on a Thermo Q Exactive HF MS in negative mode. Untargeted analysis using MAVEN software identified 4362 features (Supplementary Data File 1, Source Data File S1). These features were filtered using in-house script (Supplementary Software 1, Supplementary Software 2; see Computational methods identifying biomarkers in untargeted discovery cohort). The untargeted dataset was filtered to the most probable set of putative diagnostic signals via a significance filter (defined as features with a *p*-value less than 1.15 × 10^−5^ the Bonferroni corrected α = 0.05 significance threshold), fold change (defined as features with a minimum of a 4-fold change in average signal intensity compared to the MHB medium), and by minimum absolute intensity (defined as one or more groups showing a mean signal over 20,000 arbitrary intensity units). A total of 533 putative markers passed this filtering step (Supplementary Data File 2). These signals were clustered into 210 groups on the basis of retention times, known adduct/fragment masses, and covariance of signal intensities across replicates. The most likely parent ion was then identified from each cluster based on signal intensities (Supplementary Data File 3). Data clustering and parent ion identification were completed using our in-house software tools (see Supplementary Software 1 and Supplementary Software 2). Putative metabolite assignments were made via the Madison Metabolomics Consortium Database (MMCD)^39^ and Human Metabolome Database^40^, and select biomarkers were confirmed by analyzing the chromatographic retention times and MS/MS fragmentation patterns of commercial standards in comparison to the signals observed in the microbial extracts. Metabolite assignments were further validated by demonstrating a concentration-dependent increase in signal intensities when these standards were added to the corresponding microbial extract (Supplementary Data Fig. 1; Supplementary Data File 4).

**Fig. 2:**
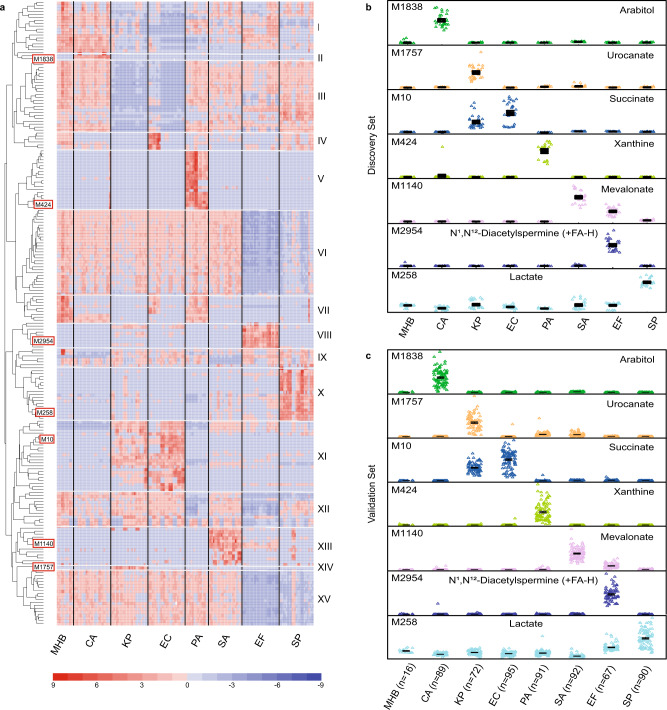
Metabolic preference assay (MPA) of seven prevalent pathogens responsible for bloodstream infections. **a** Heatmap of the top 210 putative biomarkers after a 4 h incubation period. Putative biomarkers were selected from 4362 features observed in LC-MS spectra of the discovery dataset (three independent experiments (*n* = 3), where three clinical isolates (2 for PA) of each target species (*n* = 7) were incubated independently in triplicate (*n* = 3) and analyzed; Supplementary Data Files 1–3). Biomarkers were filtered based on variance significance, signal intensity thresholds and fold change thresholds, clustered into groups based on co-retention, m/z shift relative to known fragments/adducts, and covariance, and parent ions were assigned. Putative metabolite assignments were then assigned to each via database searching^39,40^ and these assignments were confirmed by matching the MS/MS fragmentation patterns and chromatographic retention times to those observed of commercially purchased standards (Supplementary Fig. 1; Supplementary Data File 4). Putative marker numbers (M) in panel A correspond to the metabolites identified in panels **b** and **c**. **b** Discovery dataset and **c** validation dataset consisting of 596 clinical isolates (Supplementary Data File 5) demonstrate that top seven biomarkers that can robustly differentiate between the seven species studied. MHB Mueller Hinton broth with 10% blood, CA *Candida albicans,* KP *Klebsiella pneumonia,* EC *Escherichia coli*, PA *Pseudomonas aeruginosa,* SA *Staphylococcus aureus*, EF *Enterococcus faecalis,* SP *Streptococcus pneumonia*.

Species-dependent consumption or production of 210 putative makers identified in our discovery dataset robustly differentiate between the seven target species (Fig. 2a). Although the overall pattern of markers was similar between closely related microbes (i.e., *K. pneumoniae* and *E. coli*), the metabolic patterns we observed were species-specific. Remarkably, just seven production biomarkers were sufficient to distinguish between the target pathogens and acted as binary predictors of each species in our discovery datasets (Fig. 2b). Specifically, arabitol, xanthine, and N^1^,N^12^-diacetylspermine (which ionizes as a formic acid adduct) were exclusively produced by *C. albicans*, *P. aeruginosa*, and *E. faecalis*, respectively. Both *K. pneumoniae* and *E. coli* produced succinate, but the latter did not produce urocanate. Mevalonate was produced by *S. aureus*, and to a lesser extent *E. faecalis*, but unlike *E. faecalis*, *S. aureus* did not produce N^1^,N^12^-diacetylspermine. Lactate was produced by *S. pneumoniae*, and to a lesser extent, *E. faecalis*. With the exception of arabitol production by *C. albicans*^41^ and fermentative succinate production by *E. coli* and *K. pneumoniae*^42,43^, secretion of the species-specific biomarkers mentioned above has not been previously identified.

To assess the diagnostic robustness of our seven candidate biomarkers, we quantified the levels of each of these metabolites in an independent validation cohort of 596 clinical isolates collected from bloodstream infections. The metabolic phenotypes observed in each of these isolates were analyzed by MPA and each of the 210 features identified in the discovery dataset were re-evaluated. The validation dataset showed that 203 of 210 markers had significant differences by one-way ANOVA even when using the stringent Bonferroni correction applied to the original discovery dataset (*p*-values <1.15 × 10^−5^; see Statistical analysis of biomarkers in species ID validation cohort and Supplementary Data File 5). Moreover, the seven select metabolites we prioritized for differentiating species followed the same species-specific profiles we observed in the discovery dataset (Fig. 2c) and these differences were highly significant (*p*-values ranging from 1.6 × 10^−136^ to 1.1 × 10^−279^ by one-way ANOVA). Significant biomarker/species associations were identified via pairwise post-hoc comparisons using Tukey-Kramer HSQ with significant associations defined as *p*-values less than 0.01. All of our top seven biomarkers were significantly linked to one or more species via this post-hoc test (Supplementary Data File 5). Since microbes were cultured from frozen stocks ex vivo, no associations between microbial biomarkers and patent demographics were expected. However, putative demographic/biomarker associations were formally tested by collecting patient information from each of the original BSI samples (age, sex) and any possible associations with the biomarkers were tested independently via one-way ANOVA. As expected, among the 203 significant biomarkers we identified, none were significantly linked to patient age or sex (see Statistical analysis of biomarkers in species ID validation cohort; Supplementary Data File 5). In summary, two independent cohorts of samples demonstrated that metabolic profiles can robustly differentiate between species and a select set of seven metabolites is sufficient for identifying common BSI pathogens.

To assess the quantitative reliability of our MPA assay we prepared mixtures of chemical standards to serve as calibration reference standards and quality control samples. These standard mixtures were then analyzed alongside 864 clinical samples interspersed at an interval of one standard set for each batch of 96 clinical samples (*N* = 945 total samples). Concentrations of each metabolite were then computed using the calibration reference mixtures and the error associated with each quality control sample was then calculated as the root mean square error (*RMSE*). These error rates ranged from 8.9 to 30.3% (RMSE/[actual] × 100) across metabolites (Supplementary Data File 6). In comparison, the average fold changes for the same biomarkers ranged from 64- to 215-fold change relative to our control samples (uninoculated MHB). Consequently, our quantitative error is less than ≤0.6% of the species-linked phenotypes and thus does not appreciably affect the performance of our MPA assay. Notably, the addition of 10% blood to the medium, irrespective of the donor (*n* = 20), had negligible effects on metabolite profiles when compared to seeded culture, demonstrating that these putative biomarkers were pathogen-specific, and did not reflect blood metabolism or donor-specific metabolite carryover (Supplementary Fig. 2; Supplementary Data File 7). Together, these data show that the changing patterns of metabolites present in microbial cultures can be harnessed as a robust diagnostic tool for identifying bloodstream pathogens.

### Rapid antibiotic susceptibility testing via the metabolic inhibition assay

One major advantage of using metabolomics for microbial diagnostics is that metabolism is a sensitive reporter of cell physiology. Nutritional precursors are converted into waste products at rates that are many orders of magnitude faster than microbial growth. Moreover, these processes are dramatically altered, or halted completely, when cells are exposed to toxic substances. Consequently, metabolomics approaches offer a unique opportunity to empirically assess antibiotic sensitivity in a fraction of the time that is required by current growth-based AST approaches. Herein, we evaluate the practicality of using a MPA-based metabolic inhibition assay (MIA) as a diagnostic platform for quantifying antimicrobial susceptibilities.

**Fig. 3:**
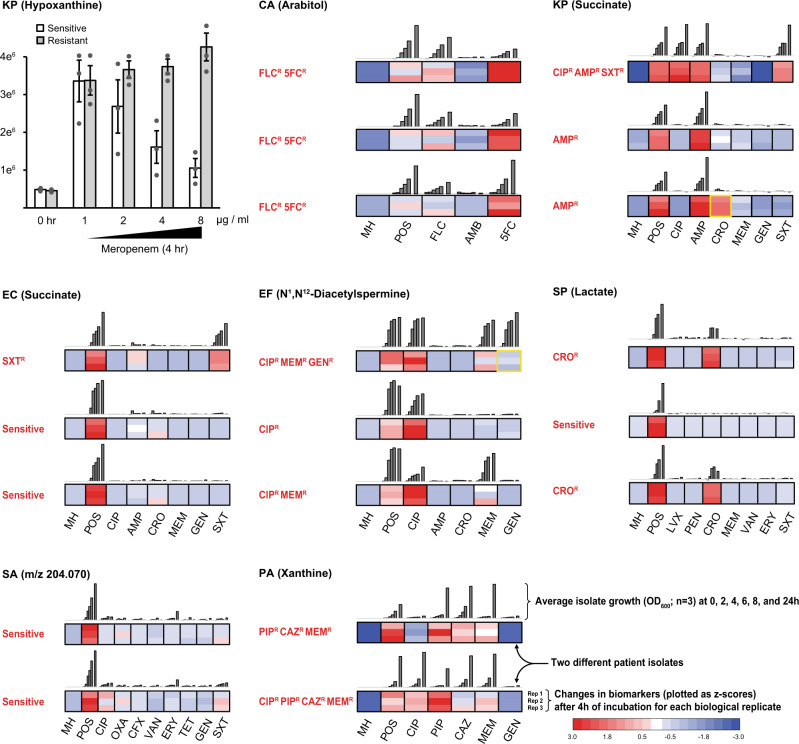
Metabolic inhibition assay (MIA) for assessing antimicrobial susceptibility. Concentrations of select biomarkers produced by a representative cohort of clinical isolates (7 species, 2–3 isolates each, performed in triplicate) were quantified after a 4 h incubation in the presence of antimicrobials. The top left panel demonstrates the MIA concept where a concentration-dependent decrease in hypoxanthine production in response to meropenem dose is observed, an effect that is not observed in drug-sensitive isolates (Data are presented as mean values ± SD). Remaining panels show the metabolic responses of pathogens exposed to antimicrobials prepared at the minimum inhibitory CLSI breakpoints differentiating sensitive versus intermediate resistant isolates (See Supplementary Data File 8)^22^. The empirically determined susceptibility profiles, as measured by VITEK 2, are reported in red text alongside each panel, and were confirmed independently by growth assays (gray bars). All MIA-determined susceptibility profiles matched those reported by the VITEK 2 with only two exceptions (highlighted in yellow boxes), resulting in a 2% false discovery rate for the assay (1% very major error and 1% major error by CLSI guidelines). (MIC in µg/mL): FLC fluconazole (2), AMB amphotericin B (2), 5FC 5-flucytosine (0.5), CRO Ceftriaxone (0.5 and 1 for *S. pneumoniae* and *K. pneumoniae*/*E. coli*, respectively), CIP ciprofloxacin (1), MEM meropenem (0.25, 1, and 2 for *S. pneumoniae*, *K. pneumoniae*/*E. coli*, and *P. aeruginosa*, respectively), GEN gentamicin (4; 500 only for *E. faecalis*), AMP ampicillin (8), SXT trimethoprim-sulfamethoxazole (2/38), CAZ ceftazidime (8), PIP piperacillin (16), LVX levofloxacin (2), PEN penicillin (0.06), ERY erythromycin (0.25 and 0.5 for *S. pneumoniae* and *S. aureus*, respectively), OXA oxacillin (2), VAN vancomycin (1, 2, and 4 for *S. pneumoniae*, *S. aureus*, and *E. faecalis*, respectively), CFX cefazolin (4), TET tetracycline (4).

MIA was accomplished by monitoring changes in the metabolic composition of microbial culture supernatants after a 4 h incubation period with and without antimicrobials. Microbes were seeded into MHB medium at 10% of a 0.5 McFarland (to a final OD_600_ of ~0.015) and metabolomics analyses were conducted using the same method used for general MPA testing. The metabolic concept underpinning MIA is illustrated in the top left panel of Fig. 3: drug-sensitive strains of *K. pneumoniae* show an incremental reduction in hypoxanthine production proportional to meropenem concentrations whereas resistant strains remain unaffected within the clinically relevant antibiotic concentrations. Similar antibiotic-induced metabolic perturbations were observed in all of the target pathogens when isolates were exposed to minimum inhibitory concentrations of commonly prescribed antimicrobials (Fig. 3; Supplementary Data File 8).

To assess MIA as a potential clinical tool, three patient isolates for each target pathogen (two for *S. aureus* and *P. aeruginosa*) were analyzed. Antifungals (azoles, polyenes, and antimetabolites) were tested for *C. albicans*. Both bactericidal (penicillins, cephalosporins, carbapenems, glycopeptides, aminoglycosides, fluoroquinolones and trimethoprim-sulfamethoxazole) and bacteriostatic (macrolides and tetracyclines) antibiotic classes were evaluated. Antimicrobial sensitivity profiles determined by MIA were consistent with 98% of the profiles observed in traditional microbial growth assays. The assays were consistent across all antimicrobial’s mechanisms of action (Fig. 3; Supplementary Data File 8). For example, succinate production by ampicillin (AMP) and trimethoprim/sulfamethoxazole (SXT) resistant *E. coli* was comparable when the strain was incubated in the presence of AMP, SXT, or in the absence of antibiotics. However, succinate production was significantly lower (*p* < 0.01 for all pairwise comparisons) when the strain was grown in the presence of antibiotics to which it was sensitive. In most cases, the biomarkers used to identify microbes were also useful for differentiating drug-sensitive and resistant strains (e.g., arabitol for *C. albicans*, succinate for *K. pneumoniae* and *E. coli*, N^1^,N^12^-diacetylspermine for *E. faecalis*, xanthine for *P. aeruginosa*, and lactate for *S. pneumoniae*). One exception to this trend was mevalonate, which is an excellent marker for *S. aureus* but an unreliable marker for drug resistance. Instead, an alternative compound with an *m/z* of 204.069 was identified as a more stable metric for differentiating resistant versus susceptible strains of *S. aureus*. Collectively, these data indicate that the MIA strategy can be an indicator of antimicrobial-resistant profiles.

### Performance validation of the metabolic inhibition assay using a rapid LC-MS method

To further assess the performance of the MIA, we evaluated this workflow using a larger cohort of isolates (*n* = 246) with varying antibiotic susceptibility profiles. This cohort included *E. coli* (*n* = 50), *S. aureus* (*n* = 63), *K. pneumoniae* (*n* = 35), *S. pneumoniae* (*n* = 49), *E. faecalis* (*n* = 23), and *E. faecium* (*n* = 24; Supplementary Data File 9). *E. faecium* was included in this cohort to provide *Enterococcus* isolates with antibiotic-resistant profiles since the majority of *E. faecalis* strains available were susceptible to all antibiotics tested. Isolates were challenged with commonly prescribed antibiotics at breakpoint concentrations (See Validation of the metabolic inhibition assay and metabolic inhibition calculations section for antibiotics tested). Metabolic profiles of supernatants following the 4 h MIA were analyzed using a rapid 5-minute HILIC chromatography method to maximize sample throughput. The cohort dataset was randomly divided into (i) a training set (*n* = 80 isolates) used to identify suitable AST markers and calculate their metabolic inhibition cutoffs differentiating sensitive and resistant strains and (ii) a test set (*n* = 166) used to make MIA calls and cross-validate these calls with VITEK 2 calls. In order to minimize the number of metabolites required for AST via MIA, metabolites were identified that could be used for multiple species. We identified glucose consumption as the most reliable indicator of antibiotic susceptibility for *S. aureus* and *Enterococcus* species; succinate production as the most reliable indicator for *E. coli*, and *K. pneumoniae* susceptibility; and nicotinate production as the most reliable indicator for *S. pneumoniae* susceptibility.

**Fig. 4:**
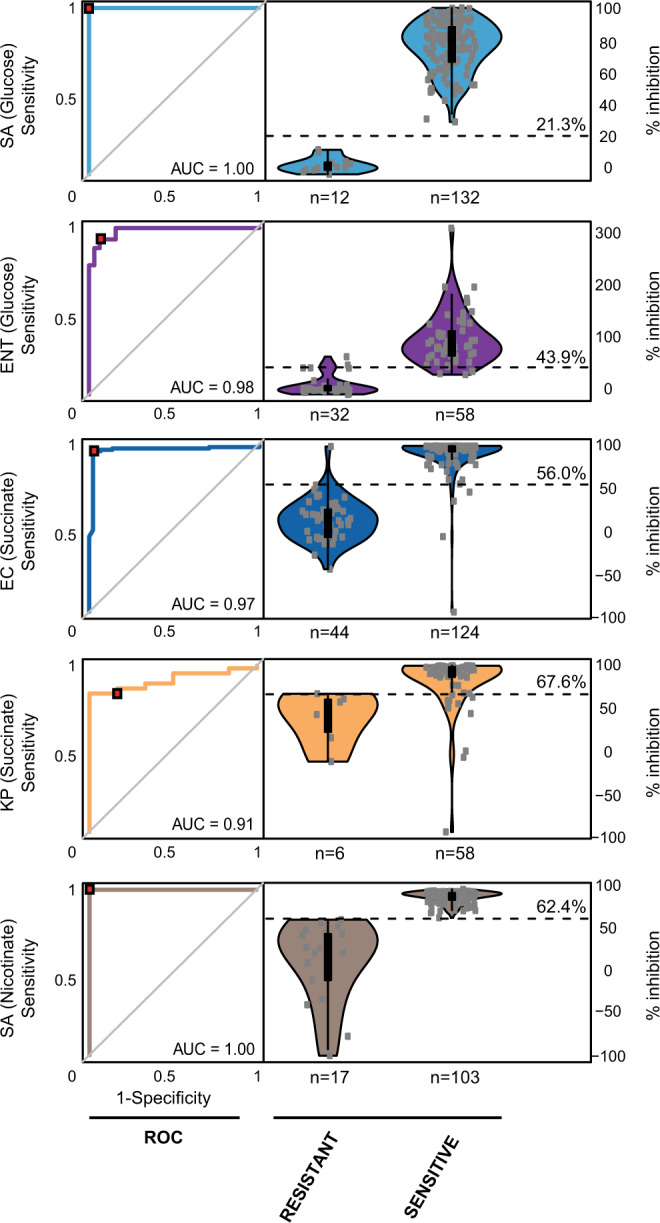
Calculated metabolic inhibition thresholds (*MIAp*, *MIAc*, %) for biomarker/species combinations used for differentiating sensitive and resistant strains using the metabolic inhibition assay. Thresholds were calculated using data from a training set containing 80 isolates using receiver operator characteristic (ROC) curves and were set to the point that co-optimized sensitivity and specificity. Given that changes in biomarkers are greater in resistant strains unaffected by antibiotics, biomarker percent inhibition values are below threshold cutoffs for resistant strains and above threshold cutoffs for sensitive strains. Metabolite levels and resistant and sensitive calls (all antibiotics combined) by VITEK 2 used to calculate thresholds are provided in Supplementary Data File 9. SA *Staphylococcus aureus*, ENT *Enterococcus* species (*E. faecalis* and *E. faecium*), EC *Escherichia coli,* KP *Klebsiella pneumonia,* SP *Streptococcus pneumonia*, AUC area under the ROC curve.

Antibiotic-induced inhibition of metabolite consumption (*MIAc*) and metabolite production (*MIAp*) was calculated using metabolite intensities from cultures incubated for 4 h in Mueller Hinton medium (see Validation of the metabolic inhibition assay and metabolic inhibition calculations section for calculations and Supplementary Data File 9). Metabolic inhibition breakpoints were then computed from receiver operating characteristic (ROC) curves for sensitive versus resistant isolates as predicted by metabolism versus predictions made via the commercial VITEK 2 antimicrobial sensitivity testing platform (Fig. 4). Metabolic breakpoints were set at thresholds that balanced maximal sensitivity and specificity. Consumption thresholds for glucose were determined to be 21.3 and 43.9% for *S. aureus* and *Enterococcus* species, respectively, while production thresholds for succinate were 56.0 and 67.6% for *E. coli* and *K. pneumonia*, respectively, and nicotinate production thresholds were 62.4% for *S. pneumonia*. The area under the ROC curve (AUC) for these metabolism-based predictions of antibiotic susceptibility ranged from 0.91 to 1 (Fig. 4).

Using the *MIAc* and *MIAp* thresholds calculated from our training set, we predicted the antimicrobial sensitivities of a test set consisting of 166 clinical isolates challenged with antibiotics. Our MIA predicted sensitivities were then compared to those generated via the commercial VITEK 2 AST testing platform (Table 1, Supplementary Data File 9). Overall, we observed a 95.2% agreement between our MIA assay and results generated by the VITEK 2 platform with species-specific predictions ranging from 90.3% for *K. pneumoniae* to 97.6% for *S. aureus*. Importantly, the major error rates (susceptible by VITEK 2 and resistant by MIA) were 3.1%, whereas the very major error rates (resistant by VITEK 2 and susceptible by MIA) were only 1.7%. Collectively, these data indicate that the MIA is a robust indicator of antimicrobial-resistance profiles.

**Table 1:** Agreement of metabolic inhibition assay results compared to VITEK 2 testing methods.

Marker (MIA threshold)	Species (*n*)	Antibiotic (ug/ml)	Sensitive called as sensitive	Resistant called as resistant	Sensitive called as resistant	Resistant called as sensitive	Correct calls per species
Glucose (>21)	SA (46)	OXA (2, 4)	29	61	0	2	359/368 (97.6%)
		CM (0.5, 4)	57	32	3	0	
		VAN (2, 8)	91	0	1	0	
		SXT (2/38, 4/76)	69	20	3	0	
		Total	246 (66.8%)	113 (30.7%)	7 (1.9%)	2 (0.5%)	
Glucose (>44)	ENT (30)	AMP (8, 16)	30	22	8	0	136/150 (90.7%)
		VAN (4, 12, 32)	58	26	2	4	
		Total	88 (58.7%)	48 (32.0%)	10 (6.7%)	4 (2.7%)	
Succinate (>56)	EC (29)	CRO (1, 4)	12	43	2	1	225/232 (97.0%)
		CIP (1, 4)	12	46	0	0	
		GEN (4, 16)	22	33	0	3	
		MER (1, 4)	54	3	0	1	
		Total	100 (43.1%)	125 (53.9%)	2 (0.9%)	5 (2.2%)	
Succinate (>68)	KP (27)	CRO (1, 4)	19	30	5	0	195/216 (90.3%)
		CIP (1, 4)	21	32	1	0	
		GEN (4, 16)	34	16	0	4	
		MER (1, 4)	29	14	11	0	
		Total	103 (47.7%)	92 (42.6%)	17 (7.9)	4 (1.9%)	
Nicotinate (>62)	SP (34)	PEN (0.06, 0.12, 2)	90	10	1	1	238/245 (97.1%)
		CRO (0.5, 2)	66	2	0	0	
		VAN (1.0, 0.25)	7	0	0	0	
		CM (0.25, 1.0)	63	0	0	5	
		Total	226 (92.2%)	12 (4.9%)	1 (0.4%)	6 (2.4%)	
Total	166		763	390	37	21	1153/1211 (95.2%)

### Metabolic-based diagnostics decrease pathogen ID and AST timelines by more than 20 h

**Fig. 5:**
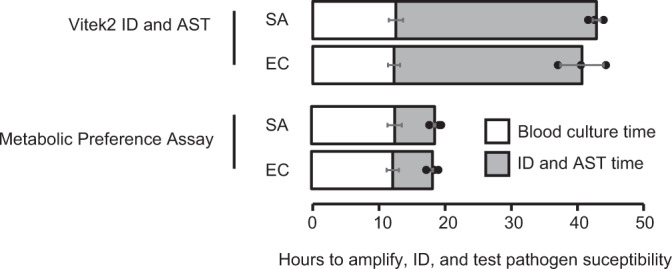
Diagnostic timelines from a head-to-head race between the VITEK 2 testing platform versus our metabolomics-based workflow. Total time to identify and perform AST using our MPA/MIA metabolomics workflow was more than 2.2-fold faster than current clinical methods, whereas identification and testing times alone were more than 4.7-fold faster. The observed 22 h decrease in testing time allows for administration of correct antimicrobial therapies, which could reduce mortality rates in septic shock patients. Three independent (*n* = 3) head-to-head races were performed between the MPA/MIA and the bioMéieux VITEK 2 platform for each organism (Supplementary Data File 10). Data are presented as mean values ± SD.

One of the primary motivations for this project is the urgent need for rapid diagnostic tools for bloodstream infections. To evaluate the potential time savings available via our MPA/MIA diagnostic workflow, we conducted three independent (*n* = 3) head-to-head races between the MPA/MIA and the bioMéieux VITEK 2 platform. Aerobic BacT/Alert bottles were seeded with 10 mL of blood and 1 mL of diluted culture containing 40–60 CFU/mL of exponential phase bacteria (*S. aureus* and *E. coli*, *n* = 3 each), and were incubated in a BacT/Alert 3D automated microbial detection system (bioMérieux) until the bottles flagged positive. One aliquot from each bottle was taken for testing on the VITEK 2, which involved plating an aliquot on blood agar plates, incubating for 18 h, picking colonies, diluting cultures according to the manufacturer’s protocols, and loading samples onto VITEK ID and AST cards. Notably, ID and AST were performed simultaneously on the VITEK. A second aliquot from each bottle was used for identifying and characterizing pathogens via the MPA/MIA workflow. For MPA/MIA analyses, media containing the most commonly tested antibiotics for each strain (CFZ, OXA, AMP, SXT, and CIP for *S. aureus*; AMP, GEN, SXT, and CIP for *E. coli*) at concentration ranges consistent with MicroScan Panels were inoculated with 0.5% of the positive blood-bacterium-BacT/Alert medium mixture. Samples were processed following the 4 h incubation period and analyzed via LC-MS using a 5-minute HILIC method. Data were analyzed in real-time using the MAVEN software package^44^. A positive growth control (medium with no antibiotic) was analyzed first to enable species identification. Subsequently, only samples incubated in the presence of antibiotics pertinent to the identified species were analyzed in order to minimize MS analysis time. Our MIA results agreed with CLSI MIC breakpoints validating that the process can be performed directly from BacT bottles. The MPA/MIA workflow reduced total testing time by an average of 24.3 h for *S. aureus* and 22.4 h for *E. coli*, corresponding to a 2.2- and 2.3-fold decrease in total testing time, respectively (Fig. 5; Supplementary Data File 10). The time required for strain identification and antibiotic susceptibility post-blood bottle flagging alone decreased by 4.7- and 5.0-fold for *S. aureus* and *E. coli*, respectively.

## Discussion

Rapid diagnostic tools for identifying microbes and measuring antibiotic susceptibility profiles could have a significant impact on morbidity and mortality rates from bloodstream infections. Moreover, these tools could help promote antibiotic stewardship by allowing clinicians to more efficiently transition patients off of broad-spectrum antibiotics and onto more precise therapies. These significant benefits are widely recognized and have driven research into a diverse collection of molecular, chemical, and optical tests that accelerate the diagnostic pipeline^45–51^. However, the metabolomics-based strategy we introduce here has several advantages over many of these emerging technologies and may represent an exciting platform for diagnosing a wide range of infectious diseases.

All free-living microbes take up nutrients from their environment and secrete metabolic waste products to support the basic requirements of life. These metabolic boundary fluxes differ considerably across microbial taxa and are the foundation for many classical microbiology identification methods (ex. carbohydrate fermentation, starch or urea hydrolysis, citrate utilization, etc.). Though effective, these classical microbiology assays generally only investigate one metabolic phenotype at a time. The metabolic preference assay we introduce here is a modern reinterpretation of classical microbiology methods that radically expands the scope of molecules that can be traced in a single assay. Although all metabolism-based assays will have a limited ability to differentiate strains or closely related species, our data show that metabolic boundary fluxes have sufficient resolution to distinguish the major taxa seen in clinical settings. While, herein, we demonstrate differentiation of seven BSI-causing organisms, this strategy could be extended to the full spectrum of BSI pathogens as well as a wide range of other microbes. We also show that metabolic boundary fluxes are altered in response to both bacteriostatic and bactericidal antimicrobial agents. These antimicrobial-induced perturbations provide a flexible diagnostic approach that could be applied to potentially any antimicrobial agent.

The MPA/MIA diagnostic workflow we introduce here has a number of advantages as a platform for clinical diagnostics. In addition to being inherently flexible with respect to infection type, it is attractive because it can integrate ID and AST testing onto a single instrument. Moreover, the MPA/MIA workflow requires minimal sample handling, allows samples to be taken directly from the blood bottle, and does not require the subculturing of isolates. Furthermore, there are already commercially available mass spectrometers certified as medical devices (e.g., Thermo Fisher Altis), which could simplify the transition of MPA/MIA into clinical practice. In summary, our MPA/MIA workflow offers an exciting strategy for identifying and phenotyping microbial cultures and could offer a path to future clinical diagnostics.

A wide range of alternative tools is emerging that could potentially improve clinical diagnostics. Some of the most exciting tools use multiplexed PCR or other DNA-based assays to accelerate diagnostics (e.g., Biofire® FilmArray® and Verigene®^25^). These assays are attractive because they can both identify pathogens and screen for a limited number of known resistance genes. Unfortunately, the presence of resistant alleles is not a direct reporter of susceptibility. Polygenic interactions, changes in expression, novel antimicrobial efflux pumps, point mutations altering primer binding, polymorphisms modulating resistance profiles, and novel resistance mechanisms can have a profound impact on a microbe’s empirically determined antimicrobial susceptibility. Furthermore, DNA-based assays cannot determine antimicrobial breakpoints, which are critical for guiding prescribing practice. In contrast, monitoring changes in metabolite levels over time when pathogens are challenged with antibiotics (e.g., via the MIA) can provide empirically determining antimicrobial susceptibilities. Specific Reveal® (Specific Diagnostics) has recently capitalized on this principle by monitoring volatile fatty acid production of cultures (post-blood bottle flagging) using colorimetric sensor arrays, allowing for AST results in as little as 5 h^52–54^. However, BSI ID using this technology has not been validated. Perhaps the most competitive platform capable of both rapid BSI ID and AST (post-blood bottle flagging) is the Accelerate Pheno® system (Accelerate Diagnostics)^55–57^. The Accelerate PhenoTest® BC can perform ID using fluorescence in situ hybridization in as little as 2 h, and AST using morphokinetic cellular analysis using time-lapse microscopy in as little as 7 h. The one major drawback of this platform is that it can only perform one test per unit which limits its utility in high-volume diagnostic labs.

Another category of emerging technologies is tools for directly detecting whole cells. For example, the AdvanDx (OpGen) multicolor qualitative fluorescent in situ hybridization probes can be used to ID pathogens in as little as 30 min from positive blood culture bottles^58,59^. This platform is exciting but requires manual staining and interpretation by skilled personnel. Furthermore, the stains used in this assay have been limited to four major groups (*Staphylococcus*, *Enterococcus*, Gram-negative species, and *Candida*) and can exhibit some cross-reactivity between species leading to false-positive results. The T2Bacteria system (T2Biosystems) is another emerging technology that employs miniaturized magnetic resonance technology and oligonucleotide-conjugated magnetic nanosensors that hybridize with specific nucleotide sequences. The T2Biosystems platform can detect ~50% of BSI pathogens at loads as low as 1 CFU/mL in as little as 3–5 h directly from blood bottles^60–63^. The main drawback OpGen, T2Biosytems, and other cell-based microbial identification systems are that these technologies generally cannot measure antibiotic susceptibility profiles, and thus must be paired with time-consuming conventional AST workflows.

The metabolomics workflow we introduce here delivers both microbial identities and empirically-determined susceptibility profiles that align with the requirements of current clinical practice. Moreover, the high sensitivity of mass spectrometry, along with the high abundance of metabolites relative to cells or macromolecules, make the MPA/MIA workflow inherently amenable to rapid diagnostics. While only the most prevalent BSI pathogens' ID and AST metrics are reported in this proof-of-concept study, expanding this workflow to less prevalent BSI pathogens will likely result in the discovery of additional biomarkers and improved species coverage. Lastly, reducing/optimizing chromatography time and transitioning the MPA/MIA workflow from a high-resolution quadrupole orbitrap mass spectrometer to existing clinical-grade triple quadrupole mass spectrometers could support the high sample throughputs needed in real-world clinical laboratories and promote uptake in laboratories with existing platforms.

In summary, we demonstrate that this ex vivo metabolomic workflow can (i) accurately identify the most common bloodstream pathogens, (ii) empirically determine antibiotic susceptibility profiles with high accuracy, and (iii) decrease diagnostic testing timelines of bloodstream infection by more than 20 h.

## Methods

### Ethics

This study was approved by the conjoint health research ethics board (REB16-2457 and REB 17-1524).

### Experimental design

All research presented here complies with all relevant ethical regulations and has been approved by the conjoint health research ethics board (REB16-2457 and REB 17-1524). The metabolic preference assay (MPA), which measures supernatant biomarker production and consumption, was used to differentiate between seven prevalent species responsible for bloodstream infections (*Candida albicans*, *Klebsiella pneumoniae*, *Escherichia coli*, *Pseudomonas aeruginosa*, *Staphylococcus aureus*, *Enterococcus faecalis*, and *Streptococcus pneumoniae*). Strains were grown in Mueller Hinton medium for 4 h, and supernatants were analyzed by ultra-high-performance liquid chromatography (UHPLC) MS to identify potential biomarkers. For initial biomarker discovery, three independent experiments were performed (*n* = 3), where three clinical isolates (2 for PA) of each target species (*n* = 7) were incubated independently in triplicate (*n* = 3) and analyzed. Untargeted MS analysis was used to find biomarkers that were common to all three isolates of each species, and that could differentiate between the different species. The stability of top biomarkers was subsequently assessed on a large cohort of clinical isolates (*n* = 596) representing these seven species. The molecular identity of select biomarkers was verified by standard additions and MS/MS fragmentation patterns comparing standards and samples. To test the applicability of the metabolic inhibition assay (MIA) for antibiotic susceptibility testing (AST), changes in metabolite concentrations were measured for each of the original strains used for biomarker discovery (*n* = 3; *n* = 2 for *S. aureus* and *P. aeruginosa*) grown in the presence of antibiotics in triplicate. The MIA was further evaluated over a larger isolate cohort (*n* = 246) and reliable markers and threshold levels of susceptible isolates were refined. Lastly, to evaluate time savings of MPA/MIA over current state-of-the-art clinical methods, a real-time head-to-head identification (ID) and AST race were performed against the VITEK 2 platform using *E. coli* and *S. aureus* on three separate occasions (see below).

### Strains, growth, and sample preparation

Isolates used in this study were recovered from patient blood culture samples and provided as cryo stocks by Alberta Precision Laboratories (APL). Patient blood samples were also provided by APL. The protocol was approved by the Conjoint Regional Ethics Board (REB # 16–2457 and REB#17-1525, UC). All chemicals were obtained from Sigma-Aldrich (St. Louis, Mo. USA), VWR (Radnor, Pa. USA), or Fisher Scientific (Waltham, Mass. USA) unless otherwise specified. With the exception of *S. pneumoniae*, all strains were routinely grown in Mueller Hinton medium (BD Difco, Mississauga, ON, Canada). *S. pneumoniae* isolates were first revived on trypticase soy agar plates containing sheep blood (BD BBL, Mississauga, ON, Canada) and then subcultured into Mueller Hinton medium supplemented with catalase (1000 U/mL). For biomarker discovery and stability testing of top biomarkers on a large cohort of clinical isolates using the MPA, exponential phase cultures were used to seed 96-well culture plates (Corning, New York, N.Y. USA) containing Mueller Hinton medium with 10% donated human blood to a 0.5 McFarland (OD_600_ ~ 0.15 or ~7.5 × 10^7^ CFU/mL). Cultures were incubated in a humidified incubator (Heracell VIOS 250i Tri-Gas Incubator, Thermo Scientific, Waltham, Mass. USA) under a 5% CO_2_ and 21% O_2_ atmosphere for 4 h. After incubation, samples were transferred to a 96-well PCR plate (VWR), and centrifuged for 10 min for 4000 × *g* at 4 °C to remove cells. The supernatant was removed, mixed 1:1 with 100% LC-MS grade methanol, and either frozen at −80 °C for further processing or centrifuged again for 10 min at 4000 × *g* at 4 °C to remove any protein precipitate. The supernatant was then diluted 1:10 with 50% LC-MS grade methanol and analyzed using UHPLC-MS. All MIAs were performed as described above; however, cultures were seeded at a 0.05 McFarland, and no blood was added to allow for periodic growth measurements at OD_600_ (Mutiskan GO, Thermo Fisher Scientific, Waltham, Mass. USA). Antibiotics used for each species were based on the prevalence of being used for treatment. Published strain-specific minimum inhibitory concentrations (MIC) of each antibiotic were used^22^.

### Validation of the metabolic inhibition assay and metabolic inhibition calculations

MIA performance was validated using a cohort of 246 isolates with varying antibiotic susceptibility profiles. Isolates were challenged with ceftriaxone (CRO; 1 and 4 ug/mL), ciprofloxacin (CIP; 1 and 4 ug/mL), gentamicin (GEN; 4 and 16 ug/mL), and meropenem (MER; 1 and 4 ug/mL) for *E. coli* and *K. pneumoniae*, oxacillin (OXA; 2 and 4 ug/mL), chloramphenicol (CM; 0.05 and 4 ug/mL), vancomycin (VAN; 2, 8 ug/mL), and trimethoprim-sulfamethoxazole (SXT; 2/38 and 4/76 ug/mL) for *S. aureus*, ampicillin (AMP; 8 and 16 ug/ml) and VAN (4, 12, and 32 ug/mL) for *Enterococcus* species, and penicillin G (PEN 0.06, 0.12, and 2 ug/mL), CRO (0.5 and 2 ug/mL, VAN (1 ug/mL), and CM (0.25 and 1 ug/mL) for *S. pneumoniae*. During the test set evaluation of the MIA, data for 27 samples treated with vancomycin (4 plates) was excluded in the final evaluation because the antibiotic was shown to be ineffective during the production of plates on that day (data provided in Supplementary Data File 9).

The cohort dataset was randomly divided into (i) a training set (*n* = 80 isolates) used to identify suitable AST markers and calculate their metabolic inhibition cutoffs differentiating sensitive and resistant strains and (ii) a test set (*n* = 166) used to make MIA calls and cross-validate these calls with VITEK 2 calls. Metabolic inhibition for markers (in percent) was calculated as follows:

1 $${{{{{\rm{MIAc}}}}}}=\frac{\left({{{{{\rm{MH}}}}}}-{{{{{\rm{Pos}}}}}}\right)-({{{{{\rm{MH}}}}}}-{{{{{\rm{AB}}}}}})}{({{{{{\rm{MH}}}}}}-{{{{{\rm{Pos}}}}}})}\times 100$$

2 $${{{{{\rm{MIAp}}}}}}=\frac{\left({{{{{\rm{Pos}}}}}}-{{{{{\rm{MH}}}}}}\right)-({{{{{\rm{AB}}}}}}-{{{{{\rm{MH}}}}}})}{({{{{{\rm{Pos}}}}}}-{{{{{\rm{MH}}}}}})}\times 100$$

where *MIAc* = the antibiotic-induced inhibition of metabolites consumed by microbes (in percent) *MIAp* = the antibiotic-induced inhibition of metabolites produced by microbes (in percent)*MH* = the intensity of a metabolite observed in control growth medium with no bacteria *Pos* = the intensity of a metabolite observed in a microbial culture growth medium with no antibiotics *AB* = the intensity of a metabolite observed in a microbial culture growth medium with antibiotics

### Real-time ID and AST race

*E. coli* and *S. aureus* were first grown overnight on tryptic soy agar plates and diluted in saline solution to ~40–60 CFU/mL. Aerobic BacT/Alert bottles were seeded with 10 mL of blood and 1 mL of diluted culture containing 40–60 CFU/mL of exponential phase bacteria (*S. aureus* and *E. coli*, *n* = 3 each), resulting in a final cell concentration of 1–1.5 CFU/mL bottle. Bottles were immediately incubated in the BacT/Alert® automated blood culture microbial detection system. Once bottles flagged, one aliquot was used for the VITEK 2 testing pipeline (see above), and a second aliquot was used for MPA. Medium containing the most commonly tested antibiotics for each strain (CIP, OXA, SXT, CFZ, and AMP for *S. aureus*; CIP, SXT, AMP, and GEN for *E. coli*) at concentration ranges consistent with the VITEK 2 automated system were inoculated with 0.5% of the positive blood-bacterium-BacT/Alert medium mixture. Samples were processed following the 4 h incubation period and analyzed via UHPLC-MS using a 5 min HILIC-MS method. Data were analyzed on the fly using the MAVEN software packages (El-MAVEN v0.12.0). The positive control (medium with no antibiotic) was analyzed first to enable species identification. Subsequently, only samples incubated in the presence of antibiotics pertinent to the identified species were analyzed in order to minimize MS analysis time.

### Chromatography and mass spectrometry

All metabolomics data were acquired at the Calgary Metabolomics Research Facility (CMRF). Metabolite samples were resolved via a Thermo Fisher Scientific Vanquish UHPLC platform using hydrophilic interaction liquid chromatography (HILIC). Chromatographic separation was attained using a binary solvent mixture of 20 mM ammonium formate at pH 3.0 in LC-MS grade water (Solvent A) and 0.1% formic acid (% v/v) in LC-MS grade acetonitrile (Solvent B) in conjunction with a 100 × 2.1 mm Syncronis^TM^ HILIC LC column (Thermo Fisher Scientific) with a 2.1 µm particle size. For general metabolic profiling runs (15 min) the following gradient was used: 0–2 min, 100 %B; 2–7 min, 100–80 %B; 7–10 min, 80–5 %B; 10–12 min, 5% B; 12–13 min, 5–100 %B; 13–15 min, 100 %B. For expedited runs (5 min) used for evaluation of MIA over a large cohort and race experiments, the gradient was as follows: 0–0.5 min, 100 %B; 0.5–1.75 min, 100–80 %B; 1.75–3 min, 80–5 %B; 3–3.5 min, 5% B; 3.5–4 min, 5–100 %B; 4–5 min, 100%B. The flow rate used in all analyses was 600 uL/min and the sample injection volume was 2 uL. Samples were ionized by electrospray using the following conditions: spray voltage of −2000 V, sheath gas of 35 (arbitrary units), auxiliary gas of 15 (arbitrary units), sweep gas of 2 (arbitrary units), the capillary temperature of 275 °C, auxiliary gas temperature of 300 °C. Positive mode source conditions were the same except for the spray voltage being +3000 V. Data were acquired on a Thermo Scientific Q Exactive^TM^ HF (Thermo Scientific) mass spectrometer using full scan acquisitions (50–750 *m/z*) with a 240,000 resolving power, an automatic gain control target of 3e^6^, and a maximum injection time of 200 ms. All data were acquired in negative mode except for MS/MS fragmentation analysis and confirmation of N^1^,N^12^-diacetylspermine, which ionized more efficiently in positive mode. Select biomarkers were confirmed using MS/MS analysis across a range of collision energies from 10–50 eV, at 30,000 resolving power, with a 5e^4^ automatic gain control target, and an isolation window of 4 m/z, selecting for previously observed parent ions. Biomarkers were matched to standards using fragmentation spectra and retention times. N^1^,N^12^-diacetylspermine was purchased from Cayman Chemical Company (Ann Arbor, Mich. USA), and all other standards were purchased from Sigma-Aldrich. Fragmentation data were analyzed using Xcalibur 4.0.27.19 software (Thermo Scientific). All other MS analyses were conducted in MAVEN (El-MAVEN v0.12.0)^44^.

### Computational methods identifying biomarkers in the untargeted discovery cohort

Untargeted biomarkers in the preliminary dataset (7 species, 3 isolates, 9 replicates) were identified by peak picking the data in El-MAVEN v0.12.0 with a 10 ppm *m/z* window and a minimum peak intensity set to 50,000. All subsequent analyses were conducted using the R statistical software platform^64^ using in-house software tools provided (Supplementary Software 1, Supplementary Software 2, Source Data File S1). The untargeted analysis identified 4,362 signals in the mass spectra (Supplementary Data File 1). Differences in mean signal intensities between groups of microbes were ranked by *p*-value (established through one-way analysis of variance; R function aov)^65^. To minimize the computational time, putative markers were screened using an alpha threshold of *p* < 1.15 × 10^−5^ (0.05/4,362; the Bonferroni corrected alpha threshold). This prioritized set of 1864 putative makers was further reduced to 533 candidate signals using a minimum peak intensity threshold (peak area-top >20,000) and minimum fold change threshold (defined as one or more microbial groups showing >4-fold difference between the no-growth control and microbial metabolite signal). This filtered set of untargeted signals contains many non-independent signals arising from in-source fragmentation, adducts, isotopomers, and other mass spectrometry-related phenomena. These clusters of signals were collapsed using a weighted probability matrix accounting for signal covariance, co-elution, and mass difference relative known adducts/fragments/isotopes including: 0.0005 (e^−^), 1.0072 (H^+^), 18.0106 (H_2_O), 34.9689 (Cl), 38.9637 (K), 22.9898 (Na), 44.9976 (Formate), 1.0062 (H neutron), 1.0034 (C neutron), 0.9694 (N neutron), 1.0042, 2.0043 (O neutrons), and 0.9994, 1.9958, 3.9950 (S neutrons)^40,66^. These signals were then clustered into 210 groups using a weighted probability function accounting for retention times, common adduct/fragment/isotopomer masses, and covariance of signal intensities among all replicates. The most likely parent ion was selected from each cluster on the basis of signal intensity and each assignment was verified by inspecting the original MS data (Supplementary Data File 3). All of the data processing steps and software are provided (Supplementary Software 1, Supplementary Software 2, Source Data File S1). These computational methods generated a list of candidate signals which were then tentatively assigned using a combination of informatics tools (Madison Metabolomics Consortium Database^39^, and the Human Metabolome Database^40^).

### Analytical methods for identifying biomarkers

Putative biomarker assignments were validated by matching MS/MS fragmentation patterns of putative signals with the fragmentation patterns of commercially available metabolite reference compounds. Assignments were further verified by adding the commercial reference compound to the microbial extract to demonstrate co-elution and concentration-dependent increases in the target biomarker signal (Supplementary Fig. 1; Supplementary Data File 4). To ensure uniform quantitative performance, mixtures of each of our target biomarkers were prepared as both calibration reference standards for absolute quantification and as quality control monitoring samples. The quantitative stability of our target biomarkers was then assessed across a cohort of 945 samples divided into nine consecutive 96-well plates. Batch-to-batch variability of the assay was then assessed as the root mean square error between the calculated versus the know concentration for metabolites of a particular concentration (most similar to that of the peak intensity of our positive growth samples) in our quality control mixture (Supplementary Data File 6).

3 $${{{{{\rm{RMSE}}}}}}=\sqrt{\frac{\sum {({{{{{\rm{Exp}}}}}}-{{{{{\rm{Obs}}}}}})}^{2}}{{{{{{\rm{n}}}}}}}}$$

Where *RMSE* = the root mean square error between the calculated and expected concentration of a particular metabolite at a specified concentration *Exp* = expected concentration of a metabolite *Obs* = calculated concentration of a metabolite *n* = total number of values (standard runs performed)

### Statistical analysis of biomarkers in species ID validation cohort

The 210 biomarkers identified in our untargeted test cohort were cross-validated using an independent cohort of 596 microbial isolates recovered from 596 individual patients. We recovered a minimum of 67 biological replicates for each bacterial species (*E. coli*, *n* = 95; *S. aureus*, *n* = 92; *K. pneumonia*, *n* = 72; *S. pneumonia*, *n* = 90; *E. faecalis*, *n* = 67; *P. aeruginosa*, *n* = 91; and *C. albicans*, *n* = 89) along with 16 no-growth control samples. Each isolate was cultured for 4 h (see Strains, growth, and sample preparation) and our 210 candidate markers were quantified by LC-MS. No technical replicates were included in this analysis. The significance of each biomarker was evaluated using one-way ANOVA (R Statistics function aov) with eight independent treatments (MHB, CA, KP, EC, PA, SA, EF, SP; seven degrees of freedom). Of the 210 candidate biomarkers identified in our discovery dataset, 203 were found to be significant after correcting for multiple hypothesis testing using the stringent *p* < 1.15 × 10^−5^ (0.05/4362; the Bonferroni alpha correction applied to the discovery set). Furthermore using Tukey-Kramer as a post-hoc analysis test (R statistics function TukeyHSD)^67–69^, species-specific biomarkers were classified, with a confidence interval of 95% (Supplementary Data File 5). Statistical associations between the MPA biomarker signals and the original patient demographics were tested by using one-way ANOVA (R statistics function aov), and no statistical significance was found between age group and sex relative to the 203 biomarkers using the R code provided (Supplementary Data File 5). All of the raw data and R statistics code needed to reproduce these analyses are provided (Supplementary Software 1, Supplementary Software 2, Source Data File S2).

### Statistical analysis of biomarkers in antibiotic susceptibility testing validation cohort

For our MIA assay, the thresholds used to differentiate sensitive versus resistant isolates were established using receiver operator characteristic curves and breakpoints were set to equilibrate sensitivity and specificity (see Fig. 4). Once thresholds were established in training sets, microbes from independent cohorts of test samples (that were grown, processed, and analyzed independently from the training set) were then assigned species identities and resistance patterns according to the thresholds. Observed error rates were then reported for these independent test cohorts.

### Reporting summary

Further information on research design is available in the Nature Research Reporting Summary linked to this article.

## Supplementary information

Supplementary Information

Reporting Summary

Description of Additional Supplementary Files

Supplementary Software 1

Supplementary Software 2

Supplementary Data File 1

Supplementary Data File 2

Supplementary Data File 3

Supplementary Data File 4

Supplementary Data File 5

Supplementary Data File 6

Supplementary Data File 7

Supplementary Data File 8

Supplementary Data File 9

Supplementary Data File 10

## Data Availability

All data are available in the main text and provided in the Source Data File and Supplementary Data Files. Putative metabolite assignments were made using the Human Metabolome Database; https://hmdb.ca/). Source data are provided with this paper.

## Source data

Source Data File

## Code availability

All of the raw data and R statistics code needed to reproduce these analyses are provided as attachments (Supplementary Software 1, Supplementary Software 2, Source Data File S1, Source Data File S2) and are published on Zenodo (https://zenodo.org/record/6403220#.YkYXoi9730o)
