# Supplementary material for: Metabolic preference assay for rapid diagnosis of bloodstream infections
Source: Nat Commun. 2022 Apr 28;13:2332. doi: 10.1038/s41467-022-30048-6 (PMC9050716; doi:10.1038/s41467-022-30048-6)
Supplement: Supplementary file 1 — Supplementary Information [file 41467_2022_30048_MOESM1_ESM.docx]

Supplementary Information

Title: Metabolic Preference Assay for Rapid Diagnosis of Bloodstream Infections

**Author List:** Thomas Rydzak^1^, Ryan A. Groves^1^, Ruichuan Zhang^1^, Raied Aburashed^2^, Rajnigandha Pushpker^1^, Maryam Mapar^1^, and Ian A. Lewis^1*^

**Affiliations:**

^1^Department of Biological Science, University of Calgary, Calgary, AB T2N 1N4, Canada.

^2^Biomedical Engineering, University of Calgary, Calgary, AB, T2N 1N4, Canada

*Correspondence to: [Ian.Lewis2@ucalgary.ca](mailto:Ian.Lewis2@ucalgary.ca)


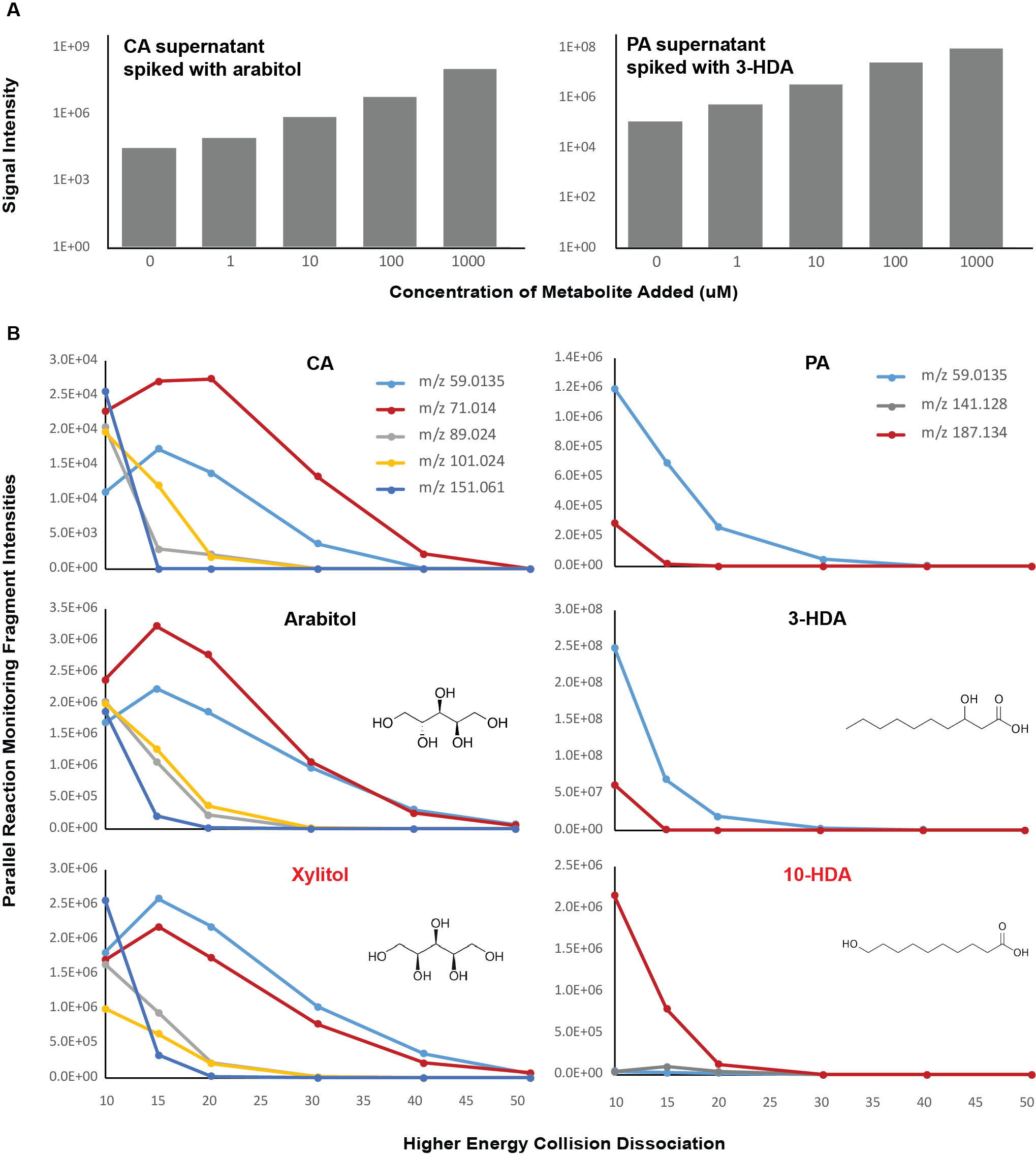


**Supplementary Fig. 1. Examples of standard addition and MS/MS fragmentation patterns used to identify biomarkers.** Standard additions were used to validate correct retention times of samples. In cases where the MS signal could arise from different isomers with similar retention times, MS/MS was used to identify the correct isomer. All standard addition and PRM data can be found in Supplementary Data File 4.


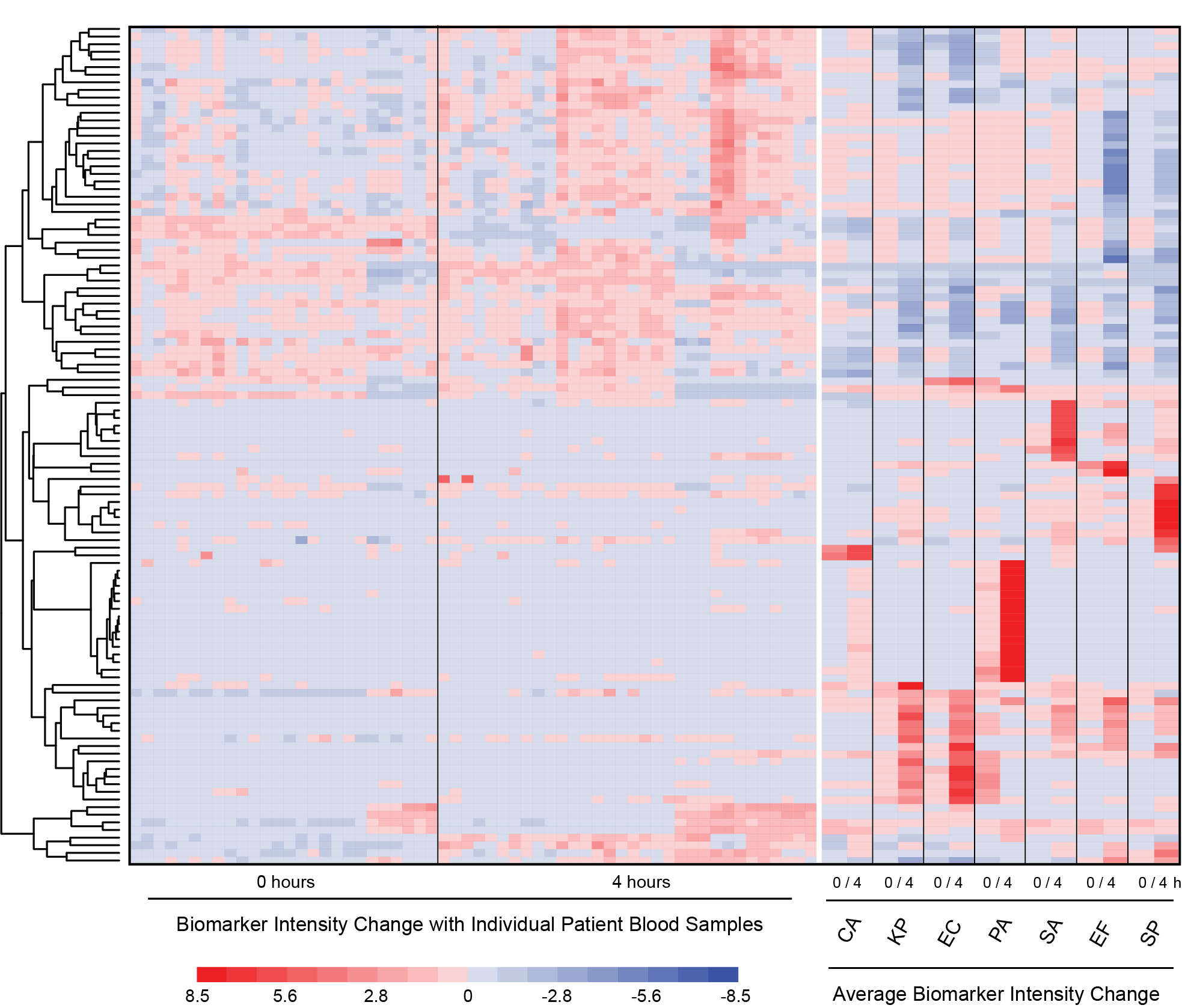


Supplementary Fig. 2. Effect of individual patient blood samples on biomarker metabolism. Individual patient blood samples (10% v:v; n = 20) were incubated in Mueller Hinton medium to assess blood carryover and metabolism of top 104 biomarkers. Changes in selected biomarker intensities was negligible when compared to average changes in biomarkers when compared to Mueller Hinton medium containing 10% blood seeded with respective pathogens. See Supplementary Data File 4 for raw data. CA, *Candida albicans*; KP, *Klebsiella pneumonia*; EC, *Escherichia coli*; PA, *Pseudomonas aeruginosa*; SA, *Staphylococcus aureus*; EF, *Enterococcus faecalis.*
