# Supplementary material for: Metabolic preference assay for rapid diagnosis of bloodstream infections
Source: Nat Commun. 2022 Apr 28;13:2332. doi: 10.1038/s41467-022-30048-6 (PMC9050716; doi:10.1038/s41467-022-30048-6)
Supplement: Supplementary file 2 — Reporting Summary [file 41467_2022_30048_MOESM2_ESM.pdf]

nature portfolio

Corresponding author(s): Ian A. Lewis

Last updated by author(s): Apr 8, 2022

Reporting Summary

Nature Portfolio wishes to improve the reproducibility of the work that we publish. This form provides structure for consistency and transparency in reporting. For further information on Nature Portfolio policies, see our Editorial Policies and the Editorial Policy Checklist.

Statistics

For all statistical analyses, confirm that the following items are present in the figure legend, table legend, main text, or Methods section.

n/a	Confirmed	
☐	☒	The exact sample size (*n*) for each experimental group/condition, given as a discrete number and unit of measurement
☐	☒	A statement on whether measurements were taken from distinct samples or whether the same sample was measured repeatedly
☐	☒	The statistical test(s) used AND whether they are one- or two-sided<br>*Only common tests should be described solely by name; describe more complex techniques in the Methods section.*
☐	☒	A description of all covariates tested
☐	☒	A description of any assumptions or corrections, such as tests of normality and adjustment for multiple comparisons
☐	☒	A full description of the statistical parameters including central tendency (e.g. means) or other basic estimates (e.g. regression coefficient) AND variation (e.g. standard deviation) or associated estimates of uncertainty (e.g. confidence intervals)
☐	☒	For null hypothesis testing, the test statistic (e.g. *F*, *t*, *r*) with confidence intervals, effect sizes, degrees of freedom and *P* value noted<br>*Give P values as exact values whenever suitable.*
☒	☐	For Bayesian analysis, information on the choice of priors and Markov chain Monte Carlo settings
☒	☐	For hierarchical and complex designs, identification of the appropriate level for tests and full reporting of outcomes
☒	☐	Estimates of effect sizes (e.g. Cohen's *d*, Pearson's *r*), indicating how they were calculated

Our web collection on statistics for biologists contains articles on many of the points above.

Software and code

Policy information about availability of computer code

Data collection	Metabolite data was acquired on a Thermo Scientific Q ExactiveTM HF (Thermo Scientific) mass spectrometer using XCalibur 4.0.27.19 software
Data analysis	Data was analyzed using El-MAVEN v0.12.0 software and statistical analysis was performed using R statistical software (mavPlot function). Statistical analysis of our biomarker data using one way ANOVA and Tukey-Kramer HMP for post hoc comparisons between groups. All code is provided in supplementary files and had been uploaded to Zenodo (https://zenodo.org/record/6403220#.YkYXoi9730o).

For manuscripts utilizing custom algorithms or software that are central to the research but not yet described in published literature, software must be made available to editors and reviewers. We strongly encourage code deposition in a community repository (e.g. GitHub). See the Nature Portfolio guidelines for submitting code & software for further information.

Data

Policy information about availability of data

All manuscripts must include a data availability statement. This statement should provide the following information, where applicable:

- Accession codes, unique identifiers, or web links for publicly available datasets
- A description of any restrictions on data availability
- For clinical datasets or third party data, please ensure that the statement adheres to our policy

All raw data is provided in the appended Source Data Files. Putative metabolite assignments were made using the Human Metabolome Database; https://hmdb.ca/).

Field-specific reporting

Please select the one below that is the best fit for your research. If you are not sure, read the appropriate sections before making your selection.

- [x] Life sciences
- [ ] Behavioural & social sciences
- [ ] Ecological, evolutionary & environmental sciences

For a reference copy of the document with all sections, see nature.com/documents/nr-reporting-summary-flat.pdf

Life sciences study design

All studies must disclose on these points even when the disclosure is negative.

Sample size	Overall sample size (isolates tested) was n = 1,146. For initial biomarker discovery, three clinical isolates (2 for PA) from each target species were analyzed in replicate (grown 9 separate times). Our power analysis (desired power set to 80%) showed that an n = 54 was sufficient to identify succinate as a biomarker, and an n = 2 to 4 was sufficient to identify all other top markers. This significance was established in a separate test cohort of 596 isolates. We constructed this larger cohort with a larger number of samples to ensure that we captured the full spectrum of microbial diversity present in a clinical population observed over a month of sample collection. To initially assess MIA as a potential clinical tool, three patient isolates for each target pathogen (2 for S. aureus and P. aeruginosa) were analyzed. The metabolic inhibition assay (MIA) was further evaluated over a larger isolate cohort (n = 246). The data from this cohort was randomly divided into a training set (n=80) to identify reliable MIA markers and threshold levels of susceptible versus resistant isolates and a test set (n = 166) to evaluate thresholds and compare results with VITEK 2 results.
Data exclusions	Mass spectrometry data was only excluded in rare cases of confirmed cross contamination of isolates. During the evaluation of the MIA over a large cohort, data for 27 samples treated with vancomycin (4 plates) was excluded in the final evaluation because the antibiotic was shown to be ineffective during the production of plates on that day (data provided in Extended Data File 9). All other antibiotic susceptibility data, including the 150 other vancomycin treated samples were included.
Replication	Biomarkers were validated using a large cohort of random clinical isolates (n = 596). MIA biomarkers were validated over a large cohort of isolates (n = 246). We followed an training/test set replication strategy wherein the test cohort was constructed from an entirely independent set of samples analyzed at a different time. As showed in the paper, the biomarkers we reported were replicated in an independent cohort.
Randomization	Clinical isolates were randomly pulled from the APL repository of >50,000 frozen isolates
Blinding	The test set (n=166) used to evaluate the MIA was blinded during data analysis.

Reporting for specific materials, systems and methods

We require information from authors about some types of materials, experimental systems and methods used in many studies. Here, indicate whether each material, system or method listed is relevant to your study. If you are not sure if a list item applies to your research, read the appropriate section before selecting a response.

Materials & experimental systems

n/a	Involved in the study
☒	☐ Antibodies
☒	☐ Eukaryotic cell lines
☒	☐ Palaeontology and archaeology
☒	☐ Animals and other organisms
☒	☐ Human research participants
☒	☐ Clinical data
☒	☐ Dual use research of concern

Methods

n/a	Involved in the study
☒	☐ ChIP-seq
☒	☐ Flow cytometry
☒	☐ MRI-based neuroimaging
