# Supplementary material for: Metabolic preference assay for rapid diagnosis of bloodstream infections
Source: Nat Commun. 2022 Apr 28;13:2332. doi: 10.1038/s41467-022-30048-6 (PMC9050716; doi:10.1038/s41467-022-30048-6)
Supplement: Supplementary file 3 — Description of Additional Supplementary Files [file 41467_2022_30048_MOESM3_ESM.pdf]

Title: Supplementary Data File 1.
Description: Untargeted MAVEN analysis of biomarkers at 0 and 4 hours. Total peak intensities for each sample are shown for each m/z and retention time (RT).

Title: Supplementary Data File 2.
Description: Filtered biomarkers at 4 hours (533 peaks). Significant biomarkers from the initial MAVEN untargeted set were first computed by analysis of variance using a Bonferroni corrected $\alpha = 0.05$. The resulting 1864 markers were then filtered to ensure that the average signal intensity for at least one organism was >20,000, and that a 4-fold change in average signal intensity compared to the Mueller Hinton Blood (MHB) medium was observed for at least one organism.

Title: Supplementary Data File 3.
Description: Parent ions of significant biomarkers (210 peaks). Peaks were clustered into 210 groups based on retention times, potential adduct/fragment masses, and covariance of signal intensities among all replicates (equally weighted) using in house R software (Supplementary Software 1, MET_LIBRARY.R; Supplementary Software 2, BSI_computation_script.R), and the parent ion, representing a real biological metabolite, was identified from each group based on highest signal intensity. Compound identifications were verified by standard additions and MS/MS (Data S4). Compounds IDs in grey have not yet been confirmed.

Title: Supplementary Data File 4.
Description: Compound identification via standard additions and MS/MS. Standards were spiked into samples at concentrations ranging from 1-1000 µM. High energy collision dissociation patterns (using collision energies from 10-50 eV) we used to confirm correct isomers.

Title: Supplementary Data File 5.
Description: Biomarker validation on clinical isolates. Biomarker consistency was assessed on 596 different clinical isolates.

Title: Supplementary Data File 6.
Description: Root mean square error of standards (observed vs expected) over 945 MS runs and average percent change in biomarker production (positive samples vs MHB negative control).

Title: Supplementary Data File 7.
Description: Blood biomarker data from 20 different patients.

Title: Supplementary Data File 8.
Description: Antibiotic susceptibility testing biomarker data.

Title: Supplementary Data File 9.
Description: Training and test data for AST validation.

Title: Supplementary Data File 10.
Description: Head-to-head race with Vitek.

Title: Supplementary Software 1.

Description: MET_LIBRARY.r – Base code/library of functions for statistical analysis.

Title: Supplementary Software 2.

Description: BSI_computation_script.r – Script to execute base code.
